# Proposal for a provisional protocol for the care and identification of dental transmission routes of COVID-19 in Latin America: A Literature review

**DOI:** 10.4317/jced.57762

**Published:** 2020-10-01

**Authors:** Frank Mayta-Tovalino, Ana Diaz-Soriano, Arnaldo Munive-Degregori, Fernando Pérez-Vargas, Silvia Luza, Rocio Bocanegra, Franco Mauricio

**Affiliations:** 1 Social Responsibility Center, Faculty of Dentistry, Universidad Nacional Mayor de San Marcos, Lima, Peru; 2 Department of Preventive and Social Stomatology, Faculty of Dentistry, Universidad Nacional Mayor de San Marcos, Lima-Peru; 3 Department of Rehabilitative Stomatology, Faculty of Dentistry, Universidad Nacional Mayor de San Marcos, Lima-Peru; 4 Department of Pediatric Stomatology, Faculty of Dentistry, Universidad Nacional Mayor de San Marcos, Lima-Peru; 5 Postgraduate Department, Faculty of Health Sciences, Universidad Científica del Sur, Lima-Peru; 6 Postgraduate Department, Faculty of Dentistry, Universidad Nacional Federico Villarreal, Lima-Peru

## Abstract

**Background:**

The new coronavirus called COVID-19 originated in the city of Wuhan, China and has currently spread to different continents, leading the World Health Organization (WHO) to declare a “pandemic”. Therefore, the aim of this review was to present a new proposal for a care protocol in Peruvian Dentistry: Provisional review of the diagnosis, treatment, epidemiological characteristics, routes of transmission and recommendations of COVID-19.

**Material and Methods:**

A search of digital scientific literature was made in the databases: Science Direct, Pubmed and Google Scholar. The Boolean operators AND, OR and NOT: “Covid-19” “Dental” “Routes of transmission. They were included Scientific articles published in English between December 2019 - March 2020.

**Results:**

Different studies were found mainly of epidemiological, observational and experimental design. On March 6, 2020, the President of the Republic of Peru confirmed and declared in Peru the beginning of the first case of coronavirus. According to reports from the Ministry of Health (MINSA) as of July 30, there are: PCR (+) 108, 299; Rapid test (+) 292, 384 positive cases and 18, 816 deceased with a lethality of 4.7%.

**Conclusions:**

Within the limitations of this literature review, the presence of the virus is inevitable in dental practice. The dentist must understand the evolution of this microorganism like all vulnerable professionals in the health sciences.

** Key words:**COVID-19, dentistry, Peru, protocol, routes of transmission.

## Introduction

On December 12, 2019, the authorities of the cities of Wuhan, China reported that there were 27 cases of an atypical viral pneumonia. What most of the affected people had in common was that they had been exposed to wild animals at the market in which it was usual to commercialize exotic birds, snakes, bats and other types of wild animals. The disease was identified from the samples of the affected patients evaluated as being caused by a novel type of coronavirus, which was temporarily named COVID-19 by the World Health Organization (WHO) and is now considered a pandemic that is rapidly infecting the entire planet (1). Initially the symptoms were diffuse, but as the casuistry of infected people increased, the following clinical symptoms were found to be typical among the patients suffering from this “new” viral disease: cough, headache, fever and in some cases hemoptysis and diarrhea (2-4).

This new COVID-19 coronavirus identified in humans has managed to mutate to be transmitted among subjects of the same species. It has been reported that saliva is one of the large viral reserves, making transmission very easy and fast by contact with the droplets that are expelled when coughing, talking, kissing, etc. Dentistry is a branch of medicine that is at high risk, since the greatest clinical activity occurs in the oral cavity with close contact with saliva, crevicular fluid, and blood, among other fluids. Indeed, the infectious potential of saliva is so high that current diagnostic methods for this new coronavirus are made by obtaining a fluid sample from the oral mucosa and oropharynx, allowing rapid, non-invasive detection of early infection by COVID-19 (5).

In a message to the nation made at 8:57 a.m on March 6, 2020, the President of the Republic of Peru confirmed the “First Case Zero “ in the city of Lima. The so-called “Case Zero” was a 25-year-old male patient with a history of having been in Spain, France and the Czech Republic several weeks previously. As of March 11, 2020, more than 100 days after the initiation of the epidemic, 86 cases have been confirmed in Peru, most being close relatives of “Case Zero”. According to the Ministry of Health of Peru (MINSA), early diagnosis is recommended with isolation of the infected patient, constant hand hygiene and avoidance of contact with infected people, among other guidelines (6,7).

As a consequence of the limited scientific evidence regarding COVID-19 infection and because the dental community is a group at high risk of contagion and dissemination of this infection, the Faculty of Dentistry of the Universidad Nacional Mayor de San Marcos (UNMSM) has expressed its commitment to Peruvian Dentistry. Therefore, for this reason the objective of this bibliographic research is to present a provisional review of the diagnosis, treatment, epidemiological characteristics, routes of transmission and recommendations of the new coronavirus (COVID-19) in Peruvian dentistry.

## Material and Methods

A search of electronic scientific literature was made in the databases: Science Direct, Pubmed and Google Scholar. The Boolean operators AND, OR and NOT: covid-19* OR dental*. They were included Scientific articles published in English between December 2019 - March 2020. Data extraction was made according the following PICO elements strategy:

P (Problem): Covid-19 viral infection

I (Intervention): Use of dental care protocol

C (Comparison): Other care protocols

O (Outcome of Interest): Maintain a minimum risk of infection in dental care

- Inclusion and exclusion criteria

Descriptive, epidemiological, experimental design studies on Covid-19 in dentistry.

Studies describing protocol of dental care

Studies describing transmission routes in dental care

Studies that are not published between December 2019 and March 2020

Studies in a language other than English

-Data extraction

The search was carried out individually by the study authors through manual consultation. The data was extracted in relation to the subject of evaluation, year of publication, methodology. A meta-analysis was not carried out because the study only presents a literature review.

-Risk of bias

Being a literature review, it is initially considered a potential risk of bias, which is stated in the conclusions of this study.

## Results

-Origin and characteristics of the covid-19

**Figure 1:**
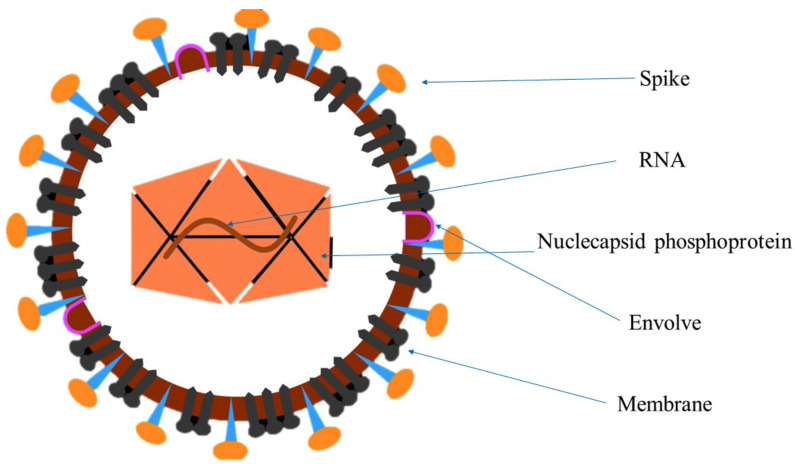
Genomic characteristics of the COVID-19.

The new coronavirus is a microorganism belonging to the β genus. This virus, which is currently known as 2019-nCoV, has an approximate diameter of 60 nm to 140 nm and is polymorphically encapsulated with oval particles. In addition, this virus has certain genetic characteristics that are inconsistent with SARSr-CoV (SARS-related coronaviruses) and MERSr-CoV (MERS-related coronaviruses). However, some recent research has shown that it has more than 85% homology to SARSr-CoV, and it seems to attach to the epithelial cells of the respiratory system (Fig. 1) (7-9).

The Coronaviridae family includes coronaviruses of the order Nidovirales, which have a single chain, RNA genome. Currently, there are four genera of coronaviruses: α-CoV, β-CoV, γ-CoV, and δ-CoV, and most of these coronaviruses mainly infect humans and some minor vertebrates (10-13).

These pathogens have a high impact on human and animal health since they generally cause respiratory and digestive diseases, and can be fatal in certain clinical situations. To date, the reported mortality rate in the city of Wuhan (China) is of around 3.8%. This city was the epicenter of today’s pandemic after the deveopment of an outbreak of an atypical picture of feverish respiratory disease due to the new COVID-19. The genetic sequence of this virus was made available to the WHO on 12 January 2020 allowing multiple laboratories in different countries to develop a specific polymerease chain reaction (PCR) diagnostic test to detect the new infection. According to the WHO, 2019-nCoV has at least 70% similarity with SARS-CoV, which is why it has been named COVID-19 (14).

According to the study by Lauer *et al.* the average incubation period for COVID-19 is approximately 5 days, similar to SARS. Assuming that infection occurs at the start of monitoring, it has been estimated that 101 out of 10 000 cases will develop symptoms after 14 days of active monitoring or quarantine. These are very important data for public health officials to establish evidence-based public prevention and control policies (15).

The genesis and virulence of COVID-19 remains uncertain. Most of the zero patients were linked to the Wuhan market in China, although, 13 of the 41 infected patients had no link to that location. Indeed, the so-called “ Patient Zero “ became ill on December 1, 2019 and had no link to the market in question, and moreover, there was no evidence of an epidemiological link between the first patient and the other infected subjects, thereby suggesting that the wild animal market was not the only place from which the virus had spread. The hypothesis that the virus infiltrated the market and spread from there was likely fabricated, however, blood tests performed in people and animals in the market could shed more light on the origin of the COVID-19 (2,16,17).

For example, because coronaviruses are microorganisms that can affect animals and humans, they are considered the predominant cause of the common cold (1,2). However, two coronaviruses are known to have greater clinical impact, and these are associated with more serious diseases: the SARS coronavirus (2002-2003) and the respiratory syndrome of the Middle East (2012) (18,19). The new COVID-19, which belongs to the B β -COV lineage, with a single-stranded positive-sense RNA encapsulated by an envelope has now been added to that list (20,21).

-Epidemiology

Epidemiologically, the new COVID-19 is a highly infectious virus, which, according to the literature, can survive approximately 2 hours in the environment and has an incubation period of approximately 4 to 8 days after infection. This virus does not distinguish between age groups, race, sex, or any other condition. However, elderly patients with several comorbidities are generally more prone to present a severe course of the disease. It is important to note that young people without a systematic history seem to remain asymptomatic. Up to now, respiratory drops have been considered the main route of transmission, although the fecal-oral route may be another source of entry for COVID-19 since nucleic acids from the virus can be present in stool samples from infected patients (22-24).

On the other hand, another source of contamination is vertical transmission between a mother and her infant. According to some studies carried out at the Wuham Children’s Hospital newborns of up to 30 hours have shown to be infected with COVID-19. In addition, some studies suggest that the ocular route should not be ignored because the lining of the eye socket can quickly become infected with droplets and other body fluids (25-27).

Zhu *et al.* (28). reported that COVID-19 has been identified as being phylogenetically closer to certain β-coronaviruses detected in some bats belonging to the Sarbecovirus subgenus of the coronaviridae family, and that this virus is 75-80% identical to SARS-CoV and 40% to MERSCoV. Therefore, it has been proposed that 2019-nCoV may share the same infection routes as SARS-CoV and MERS-CoV. In addition, some studies have shown that COVID-19 can develop more effectively in the epithelial cells of the airways, indicating that that COVID-19 employs a cell receptor human angiotensin converting enzyme 2 (hACE2) similar to that of SARS-CoV. (21,28).

**Figure 2:**
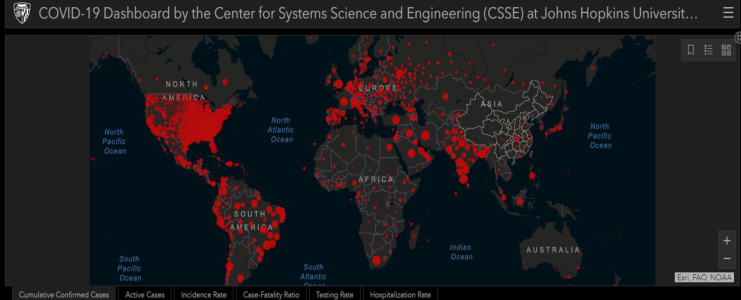
Wuhan Coronavirus COVID-19 in Peru and other global cases (07/30/2020). Provided by JHUCSSE.

The first case in Latin America was presented by a 61-year-old Brazilian man who had traveled to Italy in February 2020, where a significant outbreak of this virus is currently ongoing. The man was symptomatic on arrival home and was referred to the Albert Einstein Hospital in São Paulo, Brazil, with an RT-PCR test being positive for coronavirus. This was the first case reported in a South American country with a large population of inhabitants that has experienced previous significant outbreaks of infections declared to be world health emergencies by the WHO (Fig. 2) (29).

The first case of coronavirus in Peru was confirmed on Friday March 6, 2020. The Ministry of Health (MINSA) called for Peruvians to remain calm and confirmed that the state was prepared to face this pandemic (Table 1). It was declared that patients diagnosed with cornavirus should be transferred to 5 hospitals in the city of Lima in order to provide optimal treatment and care: Villa el Salvador Emergency Hospital, Hipólito Unanue Hospital in Agustino, Dos de Nacional Hospital May in the Cercado, Hospital de Vitarte in Ate Vitarte and Hospital Sergio Bernales de Collique (7).

**Table 1:**
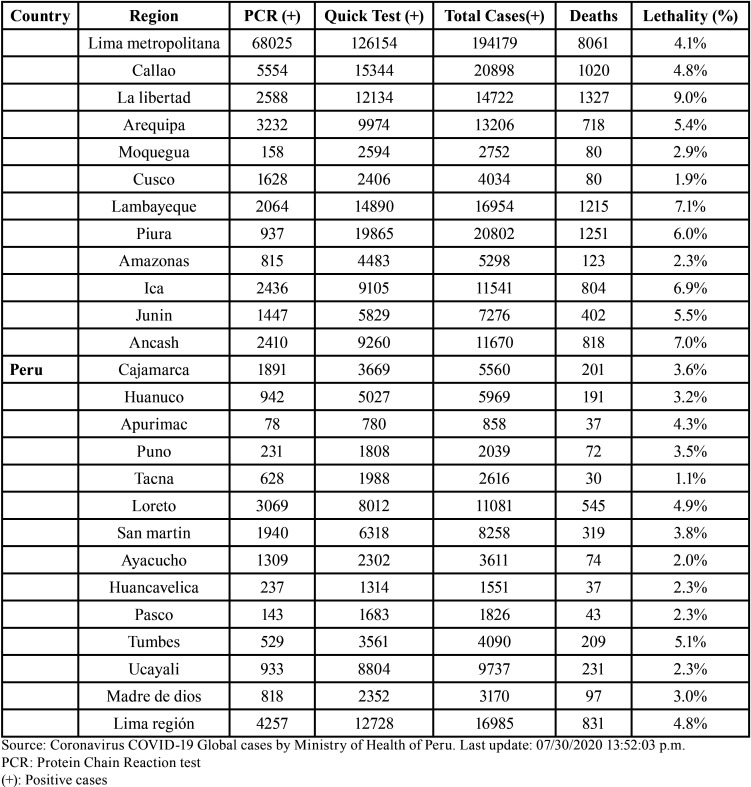
Peruvian departments affected with cases of COVID-19.

Although it is known that severe cases of COVID-19 are few compared to the total number of infected worldwide, the MINSA indicated that diagnosed cases will be monitored at home, and the Peruvian State will cover all the costs of treating the coronavirus according to the Ministerial Resolution N ° 039-2020-MINSA. Similarly, the National Institute of Health was designated as the only National Reference Laboratory authorized to diagnose COVID-19 through the corresponding biological analysis (Table 1) (7).

-Spread of covid-19 in Latin America

Within this critical situation scenario, Latin American countries have been the last to report cases of patients infected with coronavirus. On March 12, 2020, the WHO declared new COVID-19 a “pandemic” since it now affects all the continents of the world. Since the beginning of the millenium, three pathogenic outbreaks have been declared: SARS-CoV in 2002, the MERS-CoV virus in 2012 and now, the outbreak of the new COVID-19. This last microorganism develops rapidly and infectiously making it a pandemic in the world. Although the overall mortality rate is lower than that other viruses, mortality in elderly patients with systemic diseases ranges between 17-38% (18,19). To date, there is no specific treatment, and only one therapy for symptom management has been recommended (27,30-33).

**Figure 3:**
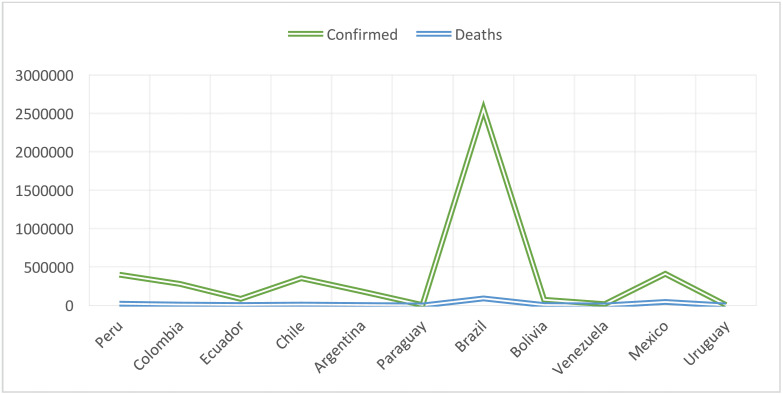
Scheme of Latin American countries affected by COVID-19. Source: Coronavirus COVID-19 Global cases by Johns Hopkins CSSE mobile version Last update: 07/30/2020 14:00:00 p.m.

Latin America is characterized by diverse climates of temperature, relative humidity and wind speed, which can be considered risk factors that must also be addressed because they can exacerbate COVID-19 transmission. Moreover, cold seasons can condition certain viruses such as SARS and influenza (34). It has been shown that the first Latin American countries presenting infection were Brazil and Mexico, leading to the question as to whether Latin American health systems are ready for this. Similar to other countries in the world, Latin American countries are still under development, and therefore, it is assumed that a pandemic of this nature can considerably affect public health (19). It is therefore essential to intensify communication efforts. In South America, there is great diversity in economic, social, and political development, among other areas. For example, some countries such as Haiti have a low development index, similar to Venezuela, which is going through a very serious social, cultural and economic crisis. Taking this into account, the impact of the new COVID-19 pandemic could be more catastrophic in these countries compared to others with more evolved economies (Table 2) (Fig. 3) (35,36).

**Table 2:**
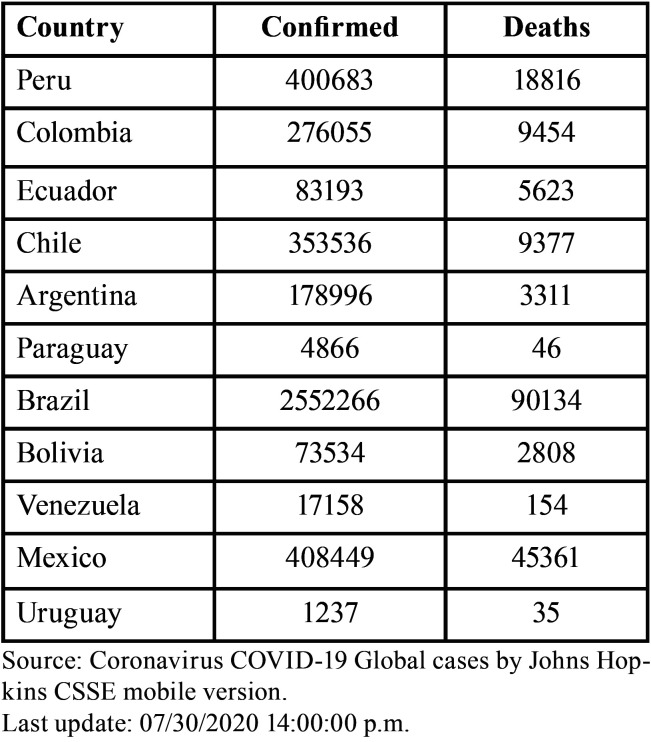
Confirmed cases in Latin America with COVID-19.

- Myths and truths

Various myths have been debunked by MINSA and the WHO in order to placate Peruvians since a lack of information to the population may exacerbate the number of newly infected patients (Table 3) (7,14).

**Table 3:**
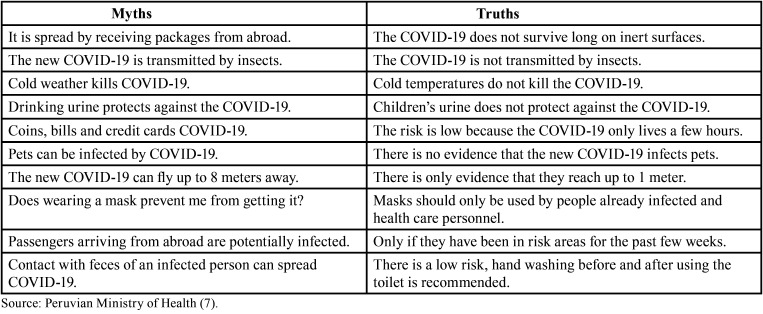
Confirmed cases in Latin America with COVID-19.

-Signs and symptoms

Patients infected with COVID-19 present with characteristic symptoms such as fever, dyspnea, dry cough, headache, nasal congestion, and other symptoms of the upper respiratory tract. However, although atypical symptoms may be present, fever remains the most typical symptom of COVID-19 infection (2,28). According to a current epidemiological survey, the symptoms of COVID-19 infection are not specific and can be confused with other pathologies because the most common symptoms are the appearance of fever, and general weakness, with some people reporting myalgia and headache among other symptoms. Diarrhea has been described in 10.6% and up to 30% of patients with SARS and MERS, respectively (21).

On the other hand, some patients present difficulty in breathing and may develop more acute clinical pictures, coagulation disorders and septic shock, while it is of note that some patients may be afebrile or asymptomatic (17,37,38). In addition, at the onset of the disease, infected children can present fever, dry cough, nasal congestion and headache, among other symptoms. However, most children generally improve after 1 week of evolution. Nevertheless, while the condition of some children can progress and evolve rapidly without major complications, the disease should be closely monitored for 1 to 3 days (17,37,38).

In relation to COVID-19 infection during pregnancy, maternal hypoxemia caused by a severe viral infection can lead to birth defects. Newborns, especially premature babies, are more likely to have insidious and nonspecific symptoms. However, most have a favorable evolution and usually recover within 1 to 2 weeks, with no deaths having been reported in this age group (6). Therefore, some criteria have been established to determine whether a possible case is infected with COVID-19 (Table 4) (8).

**Table 4:**
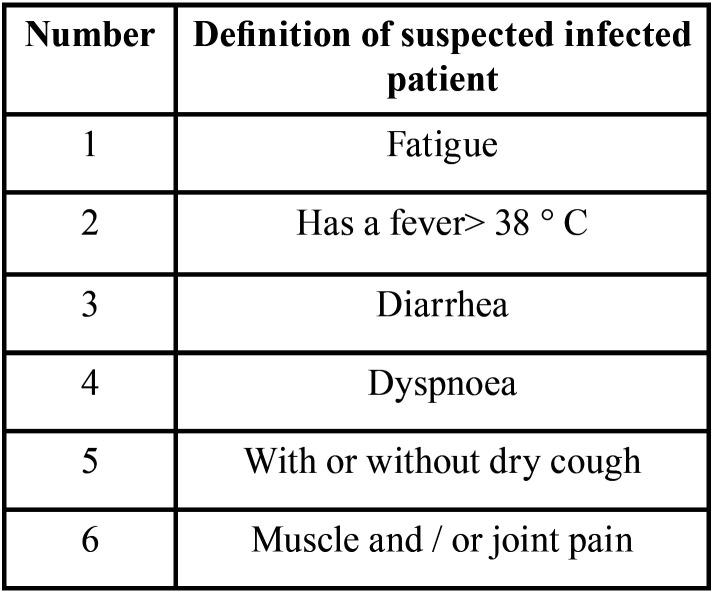
Criteria for considering a possible infected case.

-Diagnosis

The following guidelines are recommended (8):

• Clinical-physical examination: The vital functions of potentially infected patients should be monitored with special focus on respiratory sounds, and the presence of dyspnea, rhinorrhea, fever, etc.

• Imaging test: An imaging test should be performed, especially in patients with severe infection. The findings may vary with age, immunity, and stage of the disease, which must be differentiated from other viral diseases.

• Microbiological analysis: Nasopharyngeal swabs or sputum samples should be obtained for microbiological analysis. In addition, PCR assays for COVID-19 should be performed. However, the first case of COVID-19 in the United States was found to be negative in blood serum likely due to the deficient testing methods first used (17,24,39).

-Treatment (management)

• Home management: The patient should be isolated and resting at home with monitoring of vital signs (pulse, blood pressure, heart rate, respiratory rate etc.), and with adequate hydration and alimentation. In more severe cases, specialized surveillance is needed, with monitoring of organ functions (liver enzymes, urea nitrogen, myocardial enzymes, bilirubin creatinine, urine volume, etc.) (8).

• Antiviral treatment: There is currently no evidence from clinical trials that retrovirals serve as specific treatment for coronavirus in suspected or confirmed cases (8).

• Corticosteroid therapy: Glucocorticoid administration should be made with caution. For example, methylprednisolone may be indicated in a patient with an acute course of the disease, although the recommendation is weak (8). Nonetheless, the most important thing, is to isolate, track, and quarantine possible infected patients] (17,38,39).

## Discussion

Several studies are currently ongoing in an attempt to eliminate the virus. Xuentin *et al.* (40) found that hydroxychloroquine is more potent *in vitro* than chloroquine to inhibit SARS-CoV-2. Although, there is no specific recommended antiviral treatment for coronavirus, and no vaccine is currently available, treatment is symptomatic and oxygen therapy represents the main therapeutic intervention for patients with severe infection. Mechanical ventilation may be necessary in cases of respiratory failure refractory to oxygen therapy, while hemodynamic support is essential to control septic shock (27).

On the other hand, in some countries it has been suggested that the anti-HIV drug, nelfinavir, may be active against COVID-19 and could be a potentially effective drug. In addition to the previously described therapy, Chinese doctors have begun conducting clinical trials using stem cells and traditional Chinese medicine for severely ill patients. The results of these treatments will hopefully improve the evolution of patients infected with coronavirus (27-41).

As preventive treatment, the Ministry of Health (MINSA) of Peru recommends to (7):

Wash your hands frequently with soap and water (for a minimum of 20 seconds). Avoid touching your nose, mouth, and eyes. When sneezing or coughing, cover your nose and mouth with your forearm or disposable tissue. Avoid direct contact with people with respiratory problems. It should be noted that according to world casuistry, around 80% of confirmed cases infected with coronavirus recover without complications. Nevertheless, approximately 1 in 6 people can develop severe infection with difficulties in breathing, and according to the WHO, 2% of people with respiratory diseases, diabetes, heart disease or a depressed immune system can die (7).

-Dental transmission routes

**Figure 4:**
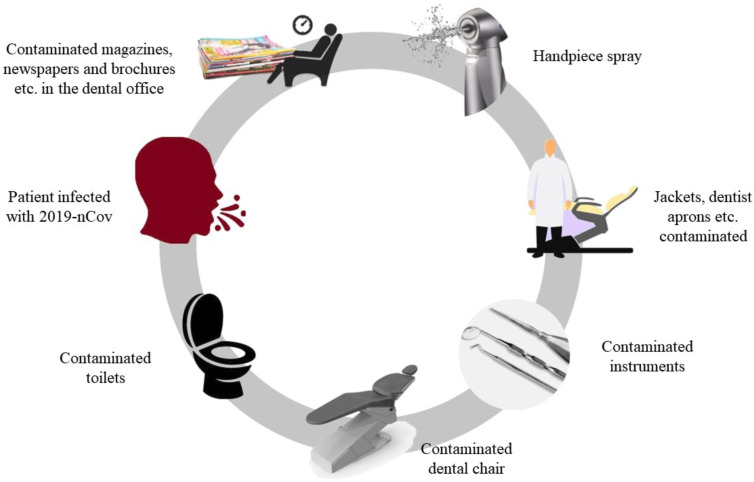
COVID-19 transmission routes in the dental office.

Since it has been shown that the COVID-19 can be spread from person to person through the micro-drops that are expelled from the airways and with knowledge of the incubation period, it has been confirmed that this virus infects the cells through the same routes as other viruses such as SARS (11). The risk of infection of COVID-19 is highly dangerous due to the ease of contagion through verbal communication, body fluids, saliva, non-sterile instruments, etc. As dentistry is a field which involves direct contact with the oral cavity, viruses can be easily transmitted in the dental environment through aerosols that are suspended in the air of the dental office, and these can enter the guest through oral, ocular and nasal mucosa (Fig. 4) (42).

•Aerosol contamination of the handpiece: Most dental units produce aerosols and drops that are highly virus-laden when caring for a carrier patient. The transmission of these drops with COVID-19 is the greatest concern in hospitals, since it is practically impossible to avoid. These droplets of saliva combined with traces of blood become a time bomb for the spread of this disease. These drops can remain in the environment and enter the airways of the respiratory tract, making the COVID-19 virus highly contagious and able to spread rapidly in dental practice.

•Constant contact with saliva: Frequent direct or indirect contact by a dental professional with contaminated human saliva, blood, dental instruments, impression materials, and dental instruments or dental office surfaces make this a direct route for the spread of the coronavirus. In addition, dental operators, assistants, and office patients can quickly become infected through the mucosa. Therefore, clinical care protocols are needed in order to avoid infection with COVID-19 through these contamination routes (4,43-49).

• Direct contact with infected people: Human coronaviruses such as SARS-CoV and MERS-CoV can persist on surfaces such as glass, metal, plastic for up to two days. Therefore, contaminated surfaces and dental instruments such as rubber cups, dam holder, trays etc. are a source of contamination. On the whole, dental treatments involve contact with saliva and this bodily fluid can contaminate the surfaces of a dental office even if patients are inactive. Moreover, it has been demonstrated that these viruses can survive hours in the environment. Thus, it is recommended to maintain the work under constant disinfection to reduce the risk of becoming infected by COVID-19. Furthermore, it is recommended to avoid contact with contaminated surfaces and instruments when proper sterilization has not been carried out in order to avoid possible cross infection among patients (4,48-49).

-Management protocol in dentistry

In its capacity as the oldest and most prestigious faculty in Peru and Latin America, the Faculty of Dentistry of the Universidad Nacional Mayor de San Marcos founded on May 12, 1551 proposes the present protocol of perfectible and primary management to avoid contagion and propagation of cases of coronavirus in Peruvian dentistry.

Dental professionals should be familiar with the development, spread and evolution of COVID-19 and should be able to identify patients with coronavirus infection and take the necessary extra protective measures that should be taken during clinical practice. To avoid COVID-19 transmission dental professionals are recommended to carry out the following infection control measures, taking into account that sprays and drops are considered the main propagation routes of COVID-19 (4,7,8,50-55).

Guidelines for the use of common protective medical products in the prevention and control of coronavirus in dental practice (7,8,14,17).

1. First: Do not accept patients with obvious signs and symptoms (Table 4) of COVID-19 but rather refer them to the respective health authorities for the proper management of active disease and avoid unnecessary spread.

2. Second: Evaluate the patient using a brief questionnaire to facilitate rapid decision making by the dentist. It is recommended to ask questions regarding possible risk factors: (a) Have you had a fever in the last two weeks? (b) Have you had respiratory problems, shortness of breath, cough, in the last two weeks? Before dental treatment, patients should be triaged to quickly identify those suspected of coronavirus. The following scenarios can be found:

In the case of fever > 38ºC, treatment must be postponed and the patient must be immediately referred to MINSA for evaluation to rule out coronavirus.

In the absence of fever (<37 ºC) patients should be treated with the biosecurity measures described below.

3.Third: The waiting room must be adequately ventilated. The time between receiving different patients should not exceed 30 minutes and crowding in the waiting room must be avoided. Office furniture and surroundings must be disinfected.

Magazines, books, brochures or any other printed material should not be made available to avoid possible cross contamination between patients.

4. Fourth: Hands of both the patient and dental operators and assistants should be washed with soap and water for at least 20 seconds and dried with a disposable paper towel before and after the care of each patient. It is also recommended to use gel based on hydroalcoholic solution at 70% on the palms of the hands for 20 seconds, rubbing well between the fingers until dry.

5. Fifth: The dentist and healthcare personnel must use barrier biosecurity protection methods that include:

• Mask: a new one mask should be used for each patient since the service life is short (1 to 2 hours) and potentially dangerous microorganisms can remain.

• Latex surgical gloves: It is recommended to use gloves for each type of treatment. For example, after the determination of the absence of potential signs and symptoms of infection during triage, a new pair of disposable gloves should be used for the treatment of the patient. Remember that glove changing is not a substitute for hand hygiene.

• Mouthwash: The use of a 0.12% chlorhexidine-based mouthwash for 60 seconds before each dental procedure will reduce microbial load.

• Protective glasses: They must be used at all times to protect the eye cavities, and thus, avoid any type of contamination. The glasses should be used before, during and after treatment. Disinfect with sodium hypochlorite or alcohol before reuse with another patient.

• Protective mask: It is recommended to use protective masks since dental procedures commonly involve contact with blood, saliva or other oral fluids. Special care should be taken when using rotating elements.

• Disposable Apron gown: It is recommended to use disposable gowns to avoid contact with saliva, blood and other fluids, which may splash onto the operator’s clothing, to thereby avoid the transfer of microorganisms home, at work or other establishments. It is strictly forbidden to go outside in clinical care workclothes. Gowns must be changed and disposed of after each patient/treatment.

• Rubber dam: It is mandatory to use this accessory, especially in the initial stages of the coronavirus pandemic, since it isolates the tooth to be treated from germs, saliva, blood and other fluids, thus reducing microbial load.

• Handpiece: A rotary instrument with an anti-retraction valve should be used since the possibility of contaminating the aerosol with viruses and other microorganisms is increased, and it has been shown that the anti-retraction system can significantly reduce the return flow of oral microorganisms.

• Disinfection and sterilization: It is mandatory to carry out the antisepsis of all personal property, stools, dental chair, among other elements, using sodium hypochlorite, hydrogen peroxide, 70% alcohol and/or other disinfectants with scientifically proven antimicrobial activity.

• Biomedical Waste Management: Eliminate waste respecting the classification colors proposed by MINSA. Sharp elements must be classified and properly separated from the other waste products. It is mandatory to have an official biomedical waste collection system with a company duly registered and authorized by MINSA.

**Figure 5:**
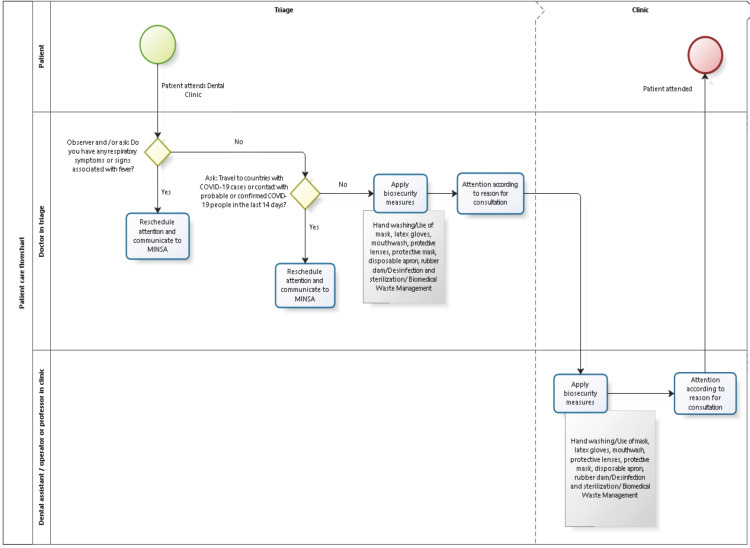
Flow chart of the new proposal of patient COVID-19 care in the dental office by the Faculty of Dentistry of the UNMSM.

Taking into account all of the above and in accordance with Ministerial Resolution No. 084-2020-MINSA, the Faculty of Dentistry of the UNMSM proposes the following flowchart for care of patients with coronavirus (Fig. 5) (7,8,14,17).

## Conclusions

In conclusion, within the limitations of this literature review the presence of the word virus in the expression of the dental professional is not new. Dentists are prepared to act quickly in the presence of bacteria, fungi, parasites and prions. The problem is to placate the population that goes to a dental service, and in this respect two paths can be taken, that of confusion or the path of knowledge. Occasionally, a virus is driven by the biological element best studied in dentistry, that is saliva, although the biochemical properties of Wharton’s secretion from the Rivinus ducts help in digestive processes On the other hand, today saliva is the vehicle leading to the shortage of masks that protect against particles of the coronavirus throughout the world.

Dental office biosecurity measures begin by questioning patients whether they have or have had fever or respiratory problems. Dental equipment is internally disinfected through the air system and water lines involving mainly the high-pressure hand piece are disinfected with sodium hypochlorite. Disinfection of all the surfaces of a dental unit is vital.

The second step is to place barriers between the patient and the operator, with special protection of the oral, nasal and ocular mucosa, being routes through which the coronavirus can be transmitted. This protection includes the use of protective glasses, masks, caps, aprons/gowns and shoe bags. Hand washing before and after treatment with plenty of soap and water reinforced with the application of more than 60% alcohol. Staff should take vitamin C and gargle with salted water to clear the virus from the throat after dental procedures. All office waste should go into a red bag as an indicator of its high viral content in order to prevent the spread of viruses, bacteria, fungi, parasites, and prions.

It is recommended to record any possible case of COVID-19 infection to serve as input and evidence of the advance of the virus to help alert as to possible mutations of this virus. The data produced by the 40 000 dentists in Peru will be our contribution to the containment of this pandemic.

## References

[1] Cheng ZJ, Shan J (2020). Novel coronavirus: where we are and what we know. Infection.

[2] Huang C, Wang Y, Li X, Ren L, Zhao J, Hu Y (2020). Clinical features of patients infected with 2019 novel coronavirus in Wuhan, China. Lancet.

[3] Wang D, Hu B, Hu C, Zhu F, Liu X, Zhang J (2020). Clinical Characteristics of 138 Hospitalized Patients With 2019 Novel Coronavirus-Infected Pneumonia in Wuhan, China. JAMA.

[4] Peng X, Xu X, Li Y, Cheng L, Zhou X, Ren B (2020). Transmission routes of 2019-nCoV and controls in dental practice. Int J Oral Sci.

[5] Sabino-Silva R, Jardim ACG, Siqueira WL (2020). Coronavirus COVID-19 impacts to dentistry and potential salivary diagnosis. Clin Oral Investig.

[6] Chen ZM, Fu JF, Shu Q, Zhu F, Liu X, Zhang J (2020). Diagnosis and treatment recommendations for pediatric respiratory infection caused by the 2019 novel coronavirus. World J Pediatr.

[7] Ministerio de Salud.

[8] Jin YH, Cai L, Cheng ZS, Cheng H, Deng T, Fan YP (2020). A rapid advice guideline for the diagnosis and treatment of 2019 novel coronavirus (2019-nCoV) infected pneumonia (standard version). Mil Med Res.

[9] (2020). Notice on the issuance of a programme for the diagnosis and treatment of novel coronavirus (2019-nCoV) infected pneumonia (Trial Version 4). General Office of National Health Committee. Office of State Administration of Traditional Chinese Medicine.

[10] Fehr AR, Perlman S (2015). Coronaviruses: an overview of their replication and pathogenesis. Methods Mol Biol.

[11] Gorbalenya A, Enjuanes L, Ziebuhr J, Snijder E (2016). Nidovirales: evolving the largest RNA virus genome. Virus Res.

[12] Nakagawa K, Lokugamage KG, Makino S (2016). in Advances in Virus Research (ed John Ziebuhr). Adv Virus Res.

[13] Fan Y, Zhao K, Shi ZL, Zhou P (2019). Bat coronaviruses in China. Viruses.

[14] Instituto Nacional de Salud.

[15] Lauer SA, Grantz KH, Bi Q, Jones FK, Zheng Q, Meredith HR (2020). The Incubation Period of Coronavirus Disease 2019 (COVID-19) From Publicly Reported Confirmed Cases: Estimation and Application. Ann Intern Med.

[16] Cohen J Wuhan seafood market may not be source of novel virus spreading globally. https://www.sciencemag.org/news/2020/01/wuhan-seafood-market-may-not-be-source-novel-virus-spreading-globally.

[17] She J, Jiang J, Ye L, Hu L, Bai C, Song Y (2020). 2019 novel coronavirus of pneumonia in Wuhan, China: emerging attack and management strategies. Clin Transl Med.

[18] Yin Y, Wunderink RG (2018). MERS, SARS and other coronaviruses as causes of pneumonia. Respirology.

[19] Chan JF, Lau SK, To KK, Cheng VCC, Woo PCY, Yuen KY (2015). Middle East respiratory syndrome coronavirus: another zoonotic betacoronavirus causing SARS-like disease. Clin Microbiol Rev.

[20] Chan JFW, Kok KH, Zhu Z, Kai-Wang To K, Yuan S, Yuen KY (2020). Genomic characterization of the 2019 novel human-pathogenic coronavirus isolated from a patient with atypical pneumonia after visiting Wuhan. Emerg Microbes Infect.

[21] Lu R, Zhao X, Li J, Niu P, Yang B, Wu H (2020). Genomic characterisation and epidemiology of 2019 novel coronavirus: implications for virus origins and receptor binding. Lancet.

[22] Wang D, Hu B, Hu C, Zhu F, Liu X, Zhang J (2020). Clinical Characteristics of 138 Hospitalized Patients With 2019 Novel Coronavirus-Infected Pneumonia in Wuhan, China. JAMA.

[23] Chen N, Zhou M, Dong X, Qu J, Gong F, Han Y (2020). Epidemiological and clinical characteristics of 99 cases of 2019 novel coronavirus pneumonia in Wuhan, China: a descriptive study. Lancet.

[24] Li Q, Guan X, Wu P, Wang X, Zhou L, Tong Y (2020). Early Transmission Dynamics in Wuhan, China, of Novel Coronavirus-Infected Pneumonia. N Engl J Med.

[25] Zhang H, Kang Z, Gong H, Xu D, Wang J, Li Z (2001). The digestive system is a potential route of 2019-nCov infection: a bioinformatics analysis based on single-cell transcriptomes. bioRxiv 2020.

[26] Lu CW, Liu XF, Jia ZF (2020). 2019-nCoV transmission through the ocular surface must not be ignored. Lancet.

[27] Han Q, Lin Q, Jin S, You L (2020). Coronavirus 2019-nCoV: A brief perspective from the front line. J Infect.

[28] Zhu N, Zhang D, Wang W, Li X, Yang B, Song J (2020). A Novel Coronavirus from Patients with Pneumonia in China, 2019. N Engl J Med.

[29] Rodriguez-Morales AJ, Gallego V, Escalera-Antezana JP, Mendez CA, Zambrano LI, Franco-Paredes C (2020). COVID-19 in Latin America: The implications of the first confirmed case in Brazil. Travel Medicine and Infectious Disease.

[30] Drosten C, Günther S, Preiser W, van der Werf S, Brodt HR, Becker S (2003). Identification of a novel coronavirus in patients with severe acute respiratory syndrome. N Engl J Med.

[31] Ksiazek TG, Erdman D, Goldsmith CS, Zaki SR, Peret T, Emery S (2003). A novel coronavirus associated with severe acute respiratory syndrome. N Engl J Med.

[32] (2020). Middle east respiratory syndrome coronavirus (MERS-CoV). WHO.

[33] Chan JFW, Kok KH, Zhu Z, Chu H, Kai-Wang To K, Yuan S (2020). Genomic characterization of the 2019 novel human-pathogenic coronavirus isolated from a patient with atypical pneumonia after visiting Wuhan. Emerg Microbes Infect.

[34] Yuan JS, Yun HM, Lan W, Wang W, Sullivan SG, Jia S (2005). A climatologic investigation of the SARS-CoV outbreak in Beijing. China Am J Infect Control.

[35] Gilbert M, Pullano G, Pinotti F, Valdano E, Poletto C, Boelle PY (2020). Preparedness and vulnerability of African countries against importations of COVID-19: a modelling study. Lancet.

[36] Paniz-Mondolfi AE, Tami A, Grillet ME, Marquez M, Hernandez-Villena J, Escalona-Rodriguez MA (2019). Resurgence of Vaccine-Preventable Diseases in Venezuela as a Regional Public Health Threat in the Americas. Emerg Infect Dis.

[37] (2020). Diagnosis and treatment of new coronavirus pneumonia (version 5). National Health Commission of the People's Republic of China.

[38] Chan JF, Yuan S, Kok KH, Kai-Wang To K, Chu H, Yang J (2020). A familial cluster of pneumonia associated with the 2019 novel coronavirus indicating person-to-person transmission: a study of a family cluster. Lancet.

[39] Holshue ML, DeBolt C, Lindquis S, Lofy KH, Wiesman J, Bruce H (2020). First case of 2019 novel coronavirus in the United States. N Engl J Med.

[40] Yao X, Ye F, Zhang M, Cui C, Huang B, Niu P (2020). In Vitro Antiviral Activity and Projection of Optimized Dosing Design of Hydroxychloroquine for the Treatment of Severe Acute Respiratory Syndrome Coronavirus 2 (SARS-CoV-2). Clin Infect Dis.

[41] Xu Z, Peng C, Shi Y, Zhu Z, Mu K, Wang X (2001). Nelfinavir was predicted to be a potential inhibitor of 2019-nCov main protease by an integrative approach combining homology modelling, molecular docking and binding free energy calculation. bioRxiv 2020.

[42] Lenkens M, de Wit H, Danser AH, Esselink AC, Horikx A, Ten Oever J (2020). Geneesmiddelen bij COVID-19 [Medication and comedication in COVID-19 patients]. Ned Tijdschr Geneeskd.

[43] To KK, Tsang OT, Chik-Yan Yip C, Chan KH, Wu TC, Man-Chun Chan J (2020). Consistent detection of 2019 novel coronavirus in saliva. Clin Infect Dis.

[44] Belser JA, Rota PA, Tumpey TM (2013). Ocular tropism of respiratory viruses. Microbiol. Mol. Biol. Rev.

[45] Rothe C, Schunk M, Sothmann P, Bretzel G, Froeschl G, Wallrauch C (2020). Transmission of 2019-nCoV Infection from an Asymptomatic Contact in Germany. N Engl J Med.

[46] Wax RS, Christian MD (2020). Practical recommendations for critical care and anesthesiology teams caring for novel coronavirus (2019-nCoV) patients. Directives concrètes à l'intention des équipes de soins intensifs et d'anesthésiologie prenant soin de patients atteints du coronavirus 2019-nCoV. Can J Anaesth.

[47] Cleveland JL, Gray SK, Harte JA, Robison VA, Moorman AC, Gooch BF (2016). Transmission of blood-borne pathogens in US dental health care settings: 2016 update. J Am Dent Assoc.

[48] Wei J, Li Y (2016). Airborne spread of infectious agents in the indoor environment. Am J Infect Control.

[49] Epidemiology Working Group (2020). Strategy and Policy Working Group for NCIP Epidemic Response, Chinese Center for Disease Control and Prevention. Zhonghua Liu Xing Bing Xue Za Zhi.

[50] Meng L, Hua F, Bian Z (2020). Coronavirus Disease 2019 (COVID-19): Emerging and Future Challenges for Dental and Oral Medicine. J Dent Res.

[51] Patel A (2020). Jernigan DB; 2019-nCoV CDC Response Team. Initial Public Health Response and Interim Clinical Guidance for the 2019 Novel Coronavirus Outbreak - United States, December 31, 2019-February 4, 2020 [published correction appears in MMWR Morb Mortal Wkly Rep. 2020 Feb 14;69(6):173]. MMWR Morb Mortal Wkly Rep.

[52] Samaranayake LP, Peiris M (2004). Severe acute respiratory syndrome and dentistry: a retrospective view. J. Am. Dent. Assoc.

[53] Hu T, Li G, Zuo Y, Zhou X (2007). Risk of hepatitis B virus transmission via dental handpieces and evaluation of an anti-suction device for prevention of transmission. Infect. Control Hosp. Epidemiol.

[54] Sabogal Á, Asencios J, Robles A, Gamboa E, Rosas J, Ríos J (2019). Epidemiological Profile of the Pathologies of the Oral Cavity in a Peruvian Population: A 9-Year Retrospective Study of 18,639 Patients. ScientificWorldJournal.

[55] Silva O, Palomino S, Robles A, Ríos J, Mayta-Tovalino F (2018). Knowledge, Attitudes, and Practices on Infection Control Measures in Stomatology Students in Lima, Peru. J Environ Public Health.

